# Anthropometry, Amplitude of Accommodation, and Spherical Equivalent Refractive Error in a Nigerian Population

**DOI:** 10.5402/2012/295613

**Published:** 2012-09-05

**Authors:** Eghosasere Iyamu, Joy Edoghogho Iyamu, Liticia Oghovwerha

**Affiliations:** ^1^Department of Optometry, Faculty of Life Sciences, University of Benin, Benin City, Nigeria; ^2^Eye Clinic, Faith Medical Complex, Benin City, Nigeria

## Abstract

*Aim*. The aim of this study was to investigate the association between anthropometry, amplitude of accommodation assessed by minus-lens to blur (AA_PUB_) and push-up to blur (AA_MLB_), and spherical equivalent refraction (SEQ). *Method*. A total of two hundred and one subjects aged between 17 and 70 years with mean age of 34.2 ± 13.3 years, consisting of 93 males and 108 females were recruited for this study. Anthropometric variables were measured with standard instruments like the free-standing rod for height, weighing balance for body weight, and body mass index (BMI) calculated. The refractive error was measured by static retinoscopy and subjective refraction. *Result*. An inverse correlation was found between age, AA_MLB_ and AA_PUB_ (*r* = −0.84, −0.81, both *P* < 0.0001). BMI increased with age (*r* = 0.32, *P* < 0.0001). There was an inverse correlation between BMI, AA_PUB_ and AA_MLB_ (*r* = −0.27, −0.25, both *P* < 0.0001), respectively. However, the association between SEQ and anthropometry was not significant (*P* > 0.05). The AA_PUB_ and AA_MLB_ decreased with age while BMI increased. AA_PUB_ and AA_MLB_ decreased with BMI, but were not affected by the SEQ. *Conclusion*. BMI increased with age while AA measured by the two methods decreased with age, and BMI increased with decreasing AA.

## 1. Introduction

The eye is one of the organs in the body that may not effectively carry out its primary function, that is, provision of clear and comfortable vision, even when it appears healthy. This is because the healthy state of the eye alone does not always guarantee provision of clear and comfortable vision for an individual within a given distance. Accommodation plays a significant role in the formation of clear retina imagery. It is considered as the ability of the eye to focus clearly for objects at various distances [1]. Refractive error is a complex interaction of the eye and its anatomical factors depending on both genetic and environmental influences, whereby light from an external object of regard is either focused before the retina (myopia), behind the retina (hyperopia), or fails to come to a single point focus on the retina (astigmatism), when accommodation is maximally relaxed [2]. A normal or desired refractive status would be when rays of light fall directly on the retina, the eye is said to be emmetropic. Refraction changes with age and occurs as a result of ocular growth after birth [3]. Amplitude of accommodation declines progressively with age beginning in the second decade of life but its symptoms are masked by an increase in depth of focus induced by accommodative miosis. Amplitude of accommodation can be stimulated by either moving a text object closer to the eyes or by placing minus lenses in front of the eyes. 

Anthropometry refers to the measurement of the height, weight, and body mass index (BMI) of an individual. Body mass index (expressed as kgms^−2^) is weight divided by height squared. Anthropometric parameters have been shown to have one effect or an other on refractive error [4–8]. To the best of our knowledge, no study has been conducted to investigate the effect of anthropometric parameters, refractive error on the amplitude of accommodation in Nigerians. This study set out to investigate the influence of anthropometric parameters on spherical equivalent refraction and the amplitude of accommodation in adult Nigerians.

## 2. Materials and Methods

This was an observational, prospective, and cross-sectional study carried out at Optometry Clinic, Department of Optometry, University of Benin, Nigeria, between October 2010 and January 2011. Informed consent was obtained from each subject after thorough explanation of the procedure and the possible result. The study was approved by the Departmental Research and Ethics Committee of the University in accordance with the tenets of Declaration of Helsinki. The subjects recruited for this study were healthy adult Nigerians. The inclusion criteria were as follows: no history of corneal trauma, surgery or pathology (infection, encroached pterygium, dystrophy, and ectasia), no history of systemic diseases (like diabetes or hypertension) or associated disorder like rheumatoid arthritis, and no history of glaucoma (open or closed angle). Only right eye measurements were taken.

The height was measured with the free standing height rod and weight using the weight scale. The BMI was obtained by dividing the weight (in kilogramme) with the square of the height (in meters) and then expressed as kgm^−2^. An extensive case history for each subject was taken to elicit the chief complaint(s) and other oculovisual complaint(s). All recordings were done with standard Snellen visual acuity notation for both far (6 m) and near (0.4 m) visual acuities. 

The amplitude of accommodation (AA) was assessed by minus-lens to blur (MLB) and push-up to blur (PUB) techniques.

### 2.1. Procedure

Monocular AA was assessed by PUB technique in which the best near VA was presented to the subject at 40 cm. The target was then gradually moved towards the subject's fixating eye until sustained blur of the print was reported. The distance between the position when the blur was sustained and the spectacle plane was taken and recorded as the near point of accommodation (NPA) in centimeters. Dividing 100 by the NPA gives the value of the AA_PUB_ (100 cm equals 1 m). For the MLB, the near point card was presented to the subject at 40 cm, while subject fixated on the 0.37 m print, minus lenses are built up until the 0.62 m print is blurred out and can barely read the 0.75 m print. The AA was taken as the sum of the stimulus to accommodation at 40 cm (2.50 D) and the minus lens power (ignoring the minus sign). For subjects that could not see the smallest print (0.37 m) line, a tentative add was placed before the eye and the process described above was repeated. The AA of the subject was recorded as the sum of the stimulus to accommodation at 40 cm (2.50 D) and the lens power after subtracting the tentative add. The resultant power was the measured AA_MLB_.

Refractive status of the participant was assessed objectively by static retinoscopy (Keeler streak retinoscope) and subjectively using American optical (AO) phoropter and full aperture trial lens set. The spherical equivalent refractive error (SEQ) was obtained by adding half the cylinder to the spherical component. SEQ ≥+0.50 D was classified as hyperopia and myopia was ≥−0.50 D.

### 2.2. Statistical Analyses

SPSS ver. 17.0 (SPSS Inc., Chicago, IL, USA) and Statgraphics Plus ver. 5.1 (Statpoint Technologies Inc., Warrenton, VA, USA) for the PC were used for data analyses and preparation of figures. Measures of spread including standardized skewness and standardized kurtosis were obtained. The measured variables were tested for normality with Kolmogorov-Smirnov *Z* test (normal distribution when the *P* value is >0.05) and when the values of standardized skewness and standardized kurtosis lie between −2 and 2. Student's *t*-test (unpaired) and Mann-Whitney *U* statistic were used to compare variables between refractive groups. Comparison of amplitude of accommodation obtained by the push-up and minus-lens to blur techniques across the age groups was done by analysis of variance (ANOVA). The relationship between variables was tested with regression analysis. Statistical significance was declared when *P* value was ≤0.05.

## 3. Results

### 3.1. Mean AA_PUB_, Effect of Age and SEQ Refraction on AA_PUB_

A total of two hundred and one (*n* = 201) subjects aged between 18 and 70 years with mean age 34.2 ± 13.3 years consisting of 93 males and 108 females were enlisted for this study.  Table 1 shows the descriptive statistics of the measured variables.

**Figure 1:**
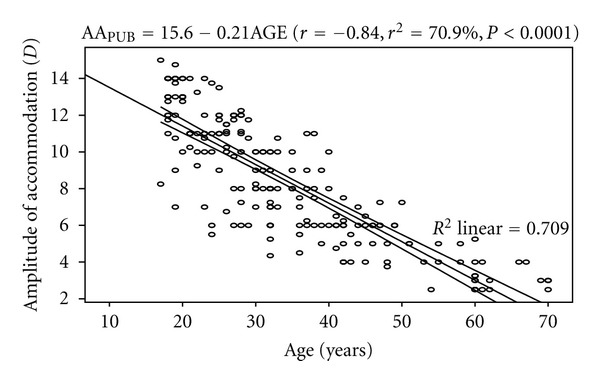
Correlation between amplitude of accommodation (push-up to blur) and age with 95% confidence interval of the linear regression line.

The mean AA was 8.48 ± 3.35 D. The regression analysis performed on AA_PUB_ and age showed a statistically significant inverse correlation (*r* = −0.84, *P* < 0.0001). The model was represented by AA_PUB_ = 15.653 − 0.21AGE. The model as fitted explains 70.9% of the variability in AA_PUB_. A 2.0 D decrease in AA was predicted for every 10-year increase in age.  Figure 1 shows the correlation between AA_PUB_ and age with the 95% confidence interval of the linear regression line.

Table 2 shows the age groups and corresponding amplitude of accommodation obtained by the two techniques.

Analysis of variance performed on the mean difference in AA_PUB_ across the age groups was statistically significant (*F* = 21.5, df = 3,197, *P* < 0.001). Post hoc (Fisher's least significant difference (LSD)) showed that the mean AA_PUB_ was significantly higher than the others. The mean differences of 4.53D (31–39 and 60–70 years), 3.13 D (31–39 and 50–59 years), 2.54 D (40–49 and 60–70 years), and 2.00 D (31–39 and 40–49 years) were statistically significant (*P* < 0.05). The correlation between AA_PUB_ and SEQ refractive error was negative but not statistically significant (*r* = −0.12, *P* = 0.08). The model was represented by the equation: AA_PUB_ = 8.42 − 0.25SEQ. From the equation, a change of 1.0 D increase in myopia will lead to a 0.25 D increase in amplitude of accommodation, while a similar change in hyperopia will result in a 0.25 D decrease in AA_PUB_. 

The difference in mean AA_PUB_ between hyperopes and myopes was statistically significant (*P* = 0.03). The amplitude of accommodation of myopes was 1.0 D higher than that of hyperopes.

### 3.2. Mean AA_MLB_, Effect of Age and SEQ Refractive Error on AA_MLB_

**Figure 2:**
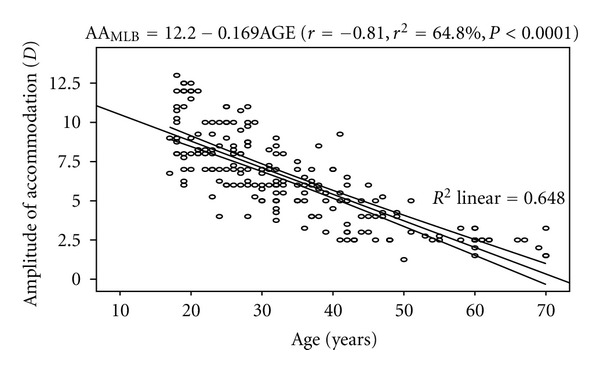
Correlation between amplitude of accommodation (minus-lens to blur) and age with 95% confidence interval of the linear regression line.

The mean AA_MLB_ was 6.36 ± 2.73 D. The regression analysis performed on AA_MLB_ and age showed a statistically significant inverse correlation (*r* = −0.81, *P* < 0.0001). The model was represented by AA_MLB_ = 11.91 − 0.162AGE. The model as fitted explains 64.8% of the variability in AA. From the equation, the amplitude of accommodation decreases by approximately 1.6 D per decade.  Figure 2 shows the correlation between amplitude of accommodation (minus-lens to blur) and age. 

The difference in mean AA_MLB_ across the age groups was statistically significant (ANOVA: *F* = 18.5, df = 3, 197, *P* < 0.001). The amplitude of accommodation of the 17–30 years age group was significantly higher than the others (*P* < 0.05). Post hoc test (Fisher's LSD) showed that the mean differences of 3.56 D (31–39 and 60–70 years), 2.89 D (31–39 and 50–59 years), and 1.82 D (31–39 and 40–49 years) were statistically significant. 

The difference in mean AA_MLB_ between hyperopes and myopes was not statistically significant (*P* = 0.26). The mean AA_MLB_ of myopes was slightly higher than that of hyperopes by 0.44 D.  Table 3 shows the effect of refractive error on anthropometry and amplitude of accommodation.

### 3.3. Correlation between Amplitude of Accommodation Measured by Minus-Lens and Push-Up to Blur Methods

**Figure 3:**
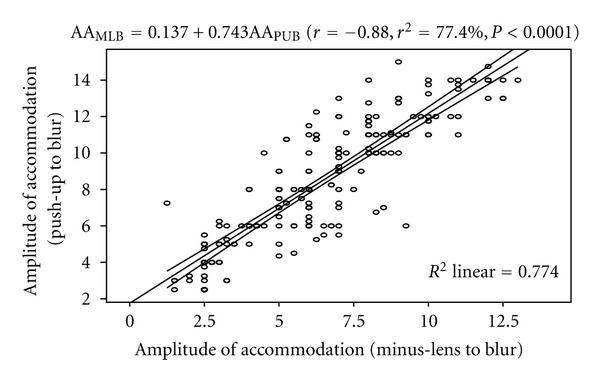
Correlation between amplitude of accommodation (push-up to blur) and amplitude of accommodation (minus-lens to blur) with the 95% confidence interval of the regression line.

The regression analysis performed on AA_PUB_ and AA_MLB_ shows a statistically significant positive correlation (*r* = 0.88, *P* < 0.0001). The linear regression model was represented by AA_MLB_ = 0.137 + 0.743AA_PUB_. The model as fitted explains 77.4% of the variability in AA_PUB_.  Figure 3 shows the correlation between AA_PUB_ and AA_MLB_. 

### 3.4. Anthropometric Parameters, Amplitude of Accommodation, SEQ Refraction, and Age

**Figure 4:**
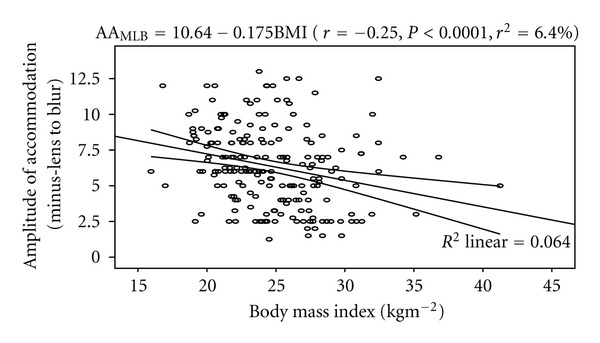
Correlation between amplitude of accommodation (minus-lens to blur) and body mass index with the 95% confidence interval of the linear regression line.

The mean height, weight, and BMI are 1.65 ± 0.11 m, 67.5 ± 11.76 kg, and 24.70 ± 3.86 kgm^−2^, respectively. Amplitude of accommodation (by minus-lens to blur and push-up to blur) was inversely correlated with height (both, *r* = −0.34, *P* < 0.0001) and weight (−0.49, −0.47, both *P* < 0.0001). Age was positively correlated with height, weight, and BMI (*r* = 0.33, 0.53, 0.32, resp., all *P* < 0.0001). Also, there was a statistically negative correlation between AA_MLB_ and BMI (*r* = −0.25, *P* < 0.0001). The model as fitted explains 6.3% of the variability in AA_MLB_. The linear regression model is represented by AA_MLB_ = 10.64 − 0.175BMI.  Figure 4 shows the correlation between AA_MLB_ and body mass index.

**Figure 5:**
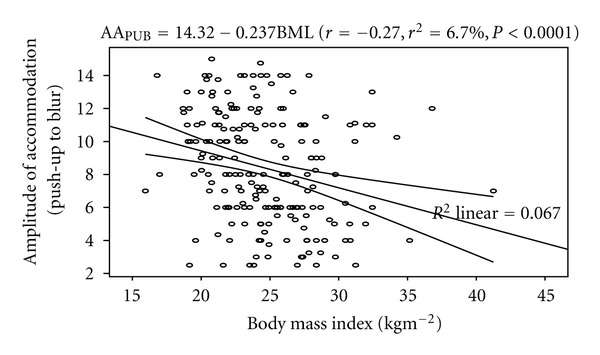
Correlation between amplitude of accommodation (push-up to blur) and body mass index with the 95% confidence interval of the linear regression line.

Similarly, a statistically significant negative correlation was found between AA_PUB_ and BMI (*r* = −0.27, *P* < 0.0001). The model as fitted explains 6.7% of the variability in AA_PUB_. The linear regression model is represented by AA_PUB_ = 14.32 *‒* 0.237BMI.  Figure 5 shows the correlation between AA_PUB_ and BMI.  Table 4 shows the Pearson's product moment correlation coefficient of the measured variables.

## 4. Discussion

Amplitude of accommodation is one of the components of near triad which defines comfort and sustainability during near visual task. In this study, the descriptive statistics of the refractive error show that the distribution was non-Gaussian (leptokurtic and negatively skewed), standardized skewness being *‒*10.00 and average refractive error of −0.25 ± 1.67 D (range, −9.50 D and +4.00 D). The overwhelming majority of our subjects (92%) had refractive error of between −2.00 and +2.00 D, and this was similar to that of Osuobeni et al. [9], as majority of their subjects had refractive error between −2.00 and +1.00 D. The difference in mean AA assessed by push-up to blur (PUB) and minus-lens to blur (MLB) was statistically significant (*P* < 0.0001). The mean AA by PUB was higher than obtained through MLB. This may be attributed to the effect of depth of focus that comes into play during PUB technique. This is consistent with the report of Grosvenor [10], who asserted that the depth of focus rather than the actual AA is measured by PUB. Depth of focus is the extent to which the image may be located in front or behind the retina and still appear to be clear [1]. The regression analysis showed a strong inverse relationship between AA_PUB_ and age, and from the equation of the model, a decrease of approximately 2.0 D in AA for every decade was predicted. A prediction of 1.7 D decrease in AA_MLB_ for every decade was made. And from the two models, by age 40 years the AA would be 5.4 and 7.2 D, respectively. This was consistent with the claim of Grosvenor [10] that AA decreases to 5.00 D or less at presbyopic age. Hofstetter [11] calculated Duane's figures and provided the model for average, maximum, and minimum AA: Ave. AA = 18.5 − 0.3AGE, Max. AA = 25.0 − 0.4AGE, and Mini. AA = 15.0 − 0.25AGE, respectively. He acknowledged that the values of AA are influenced by the technique of measurement, but they did not state the technique used to obtain the values of AA used for producing the model for the average, maximum, and minimum AA with respect to age. However, this study has gone a step further to provide a linear regression model for AA and age for the two techniques especially for the Nigerian population. The difference in mean AA assessed by MLB between hyperopes and myopes was not significant (*P* = 0.26). The MLB method measures the actual accommodative amplitude as it stimulates every amount of accommodation to come into play during measurement. On the contrary, the difference in mean AA assessed by PUB method between hyperopes and myopes was significant (*P* = 0.03), being higher for myopes. This is because the image is located in front of the retina and through PUB the depth of focus is increased reflecting as higher AA value. From the regression analysis of AA (measured by MLB and PUB) and weight a change of approximately 1.1 D and 1.4 D decrease in AA measured by MLB and PUB for every 10 kg increase in weight was predicted. For every increase of 10 cm increase in height, the AA assessed by MLB and PUB was decreased by 0.87 and 1.02 D, respectively. Heavier and taller persons have lower AA. Taller people tend to extend their arms more when reading to increase depth of focus thereby increasing the near point of accommodation with consequent decrease in amplitude of accommodation especially in the prepresbyopic and presbyopic groups. In this study it was shown that height of subjects had no significant correlation with spherical equivalent refractive error (*r* = 0.012). This was in agreement with the claims of Wong et al. [5] and Osuobeni et al. [9] who reported that the correlation between height and SEQ was not significant (*r* = −0.04 and *r* = −0.07, resp.). However, Johansen [12] reported that height varied considerably with both refractive groups (myopes and nonmyopes). Dirani et al. [13] also found that height showed a low but statistically significant inverse correlation with refractive error (*r* = −0.15, *P* < 0.01). From this study, it was shown that correlation between weight and SEQ was not significant (*r* = −0.086) and this was in line with the finding of Osuobeni et al. [9]. Similarly, Wong et al. [5] found no significant association between weight and SEQ (*r*_s_ = 0.058). Previous studies have not shown consistent association between weight and refraction [14–18]. The finding of this study also shows that the association between BMI and SEQ was not significant and this was in line with the studies of Wong et al. [5] and Osuobeni et al. [9]. From this study, it was shown that for every 10 kg and 10 kgm^−2^ difference in weight and BMI the magnitude of hyperopia was decreased by 0.10 and 0.43 D, respectively, and this was not consistent with the claims of Wong et al. [5], who reported an increase of 0.25 and 0.50 D hyperopia for the same change in weight and BMI. 

In conclusion, amplitude of accommodation measured by PUB and MLB significantly decreased with increasing age, but demonstrated no effect on the SEQ refractive error. Height and weight inversely correlated with AA assessed by both PUB and MLB. BMI was also significantly associated with AA_PUB_ and AA_MLB_. No statistically significant correlation was found between anthropometry and SEQ refractive error.

## Figures and Tables

**Table 1:** Descriptive statistics of measured physical anthropometry and other variables.

Statistics	Variables						
	Age (years)	BMI (kgm^−2^)	HT (m)	WT (kg)	SEQ (D)	AA_PUB _(D)	AA_MLB_ (D)
Count	201	201	201	201	201	201	201
Mean	34.2	24.7	1.65	67.5	−0.25	8.48	6.36
SD	13.3	3.86	0.11	11.76	1.67	3.35	2.73
Median	32	24.3	1.66	68.0	−0.50	8.00	6.00
Stnd skew	0.86	0.73	0.30	0.14	−10.00	0.038	0.26
Stnd kurt	−0.02	1.29	0.30	−0.56	11.95	−1.06	−0.66
95% CI	33.46–35.34	24.4–24.9	1.65–1.67	66.7–68.3	−0.46–0.00	8.14–8.60	6.16−6.56
*P* value (K-S)	0.08	0.48	0.15	0.25	0.03	0.10	0.29

AA_MLB_: amplitude of accommodation (minus-lens to blur); AA_PUB_: amplitude of accommodation (push-up to blur); BMI: body mass index; SD: standard deviation; Stnd skew: standardized skewness; Stnd kurt: standardized kurtosis; 95% CI: 95% confidence interval; K-S: Kolmogorov-Smirnov *Z* test; *P* value: level of significance.

**Table 2:** Age group and amplitude of accommodation measured by push-up and minus-lens to blur Methods.

Age group (years)	Amplitude of accommodation (D)		
	Count	Push-up to blur	Minus-lens to blur
17–30	83	11.28 ± 2.12	8.64 ± 2.09
31–39	47	7.77 ± 1.80	5.95 ± 1.47
40–49	37	5.78 ± 1.33	4.13 ± 1.42
50–59	13	4.64 ± 1.28	3.06 ± 0.79
60–70	21	3.24 ± 0.74	2.39 ± 0.54

**Table 3:** Summary statistics and effect of refractive error on measures of physical anthropometry and amplitude of accommodation.

	Age (years)	HT (m)	WT (kg)	BMI (kgm^−2^)	AA_PUB_ (D)	AA_MLB_ (D)	SEQ (D)
Mean (SD)							
All (*n* = 201)	34.2 (13.4)	1.66 (0.11)	67.5 (11.7)	24.7 (3.9)	8.48 (3.4)	6.36 (2.7)	−0.25 (1.67)
Hyperopes							
(*n* = 96)	35.2 (15.2)	1.70 (0.15)	68.4 (12.3)	23.2 (4.2)	7.94 (3.3)	6.11 (2.6)	+0.94 (0.68)
Myopes							
(*n* = 105)	33.7 (12.8)	1.63 (0.23)	69.8 (12.9)	25.1 (4.6)	8.95 (3.3)	6.55 (2.8)	−1.33 (1.54)
*t*-test	*t *= 0.68	*t* = 1.82	*t* = 0.96	*t* = 1.27	*t* = −2.15	*t* = −1.13	—
	*P* = 0.70	*P* = 0.12	*P* = 0.27	*P* = 0.30	*P*= 0.03	*P* = 0.26	—
M-W	*U* = 66	*U* = 110	*U* = 99	*U* = 67	*U* = 235	*U* = 66	—
	*P* = 0.62	*P* = 0.08	*P* = 0.14	*P* = 0.25	*P* = 0.02	*P* = 0.62	—
K-S test	K-S = 0.43	K-S = 1.27	K-S = 1.33	K-S = 0.87	K-S = 1.49	K-S = 0.99	K-S = 2.32
	*P* = 0.52	*P* = 0.27	*P* = 0.26	*P* = 0.43	*P* = 0.10	*P* = 0.30	*P* = 0.03
Stnd skewness							
All	0.86	0.30	0.14	0.73	0.04	0.26	10.00
Hyperopes	1.02	1.31	0.28	−1.33	−0.06	0.30	11.80
Myopes	0.79	0.42	−0.12	−1.09	−0.07	0.35	−11,86
Stnd kurtosis							
All	−0.02	0.30	−0.56	1.29	−1.06	0.66	11.95
Hyperopes	−0.05	0.60	−0.49	−1.08	−1.67	0.72	15.52
Myopes	0.16	−0.70	−0.52	0.99	−0.98	1.02	19.44

SD: standard deviation in parenthesis; *t*-test: Student's *t*-test; M-W: Mann-Whitney *U* test; K-S: Kolmogorov-Smirnov *Z* test; Stnd skewness: standardized skewness; Stnd kurtosis: standardized kurtosis; HT: Height; WT: weight; BMI: body mass index; SEQ: spherical equivalent refractive error; AAMLB: amplitude of accommodation (minus-lens to blur); AAPUB: amplitude of accommodation (push-up to blur).

**Table 4:** Pearson product moment correlation coefficient between measured variables.

	WT (kg)	HT (m)	BMI (kgm^−2^)	AA_PUB _(D)	AA_MLB_ (D)	SEQ (D)
Age (years)	0.52 (**<0.0001**)	0.31 (**<0.0001**)	0.32 (**<0.0001**)	−0.84 (**<0.0001**)	−0.81 (**<0.0001**)	0.09 (0.22)
Weight (kg)		0.55 (**<0.0001**)	0.66 (**<0.0001**)	−0.48 (**<0.0001**)	−0.48 (**<0.0001**)	−0.09 (0.23)
Height (m)			−0.24 (**<0.0001**)	−0.34 (**<0.0001**)	−0.34 (**<0.0001**)	0.01 (0.87)
BMI (kgm^−2^)				−0.27 (**<0.0001**)	−0.25 (**<0.0001**)	0.11 (0.13)
AA_PUB_ (D)					0.88 (**<0.0001**)	−0.08 (0.28)
AA_MLB _(D)						−0.05 (0.38)

First numbers are correlation coefficients; second numbers are *P* values; significant correlation coefficients are shown in bold; SEQ: spherical equivalent refractive error; BMI: Body mass index; AA_PUB_: amplitude of accommodation (push-up to blur); AA_MLB_: amplitude of accommodation (minus-lens to blur).
